# The AgeWell study of behavior change to promote health and wellbeing in later life: study protocol for a randomized controlled trial

**DOI:** 10.1186/1745-6215-13-115

**Published:** 2012-07-24

**Authors:** Linda Clare, John V Hindle, Ian R Jones, Jeanette M Thom, Sharon M Nelis, Barry Hounsome, Christopher J Whitaker

**Affiliations:** 1 School of Psychology, Bangor University, Bangor, Gwynedd, LL57 2AS, UK; 2 School of Medical Sciences, Bangor University, Bangor, UK; 3 School of Social Sciences, Bangor University, Bangor, UK; 4 School of Sports, Health and Exercise Sciences, Bangor University, Bangor, UK; 5 Centre for Health Economics and Medicines Evaluation, Bangor University, Bangor, UK; 6 North Wales Organisation for Randomised Trials in Health, Bangor University, Bangor, UK

**Keywords:** Cognitive activity, Physical activity, Social engagement, Prevention, Quality of life, Health, Diet, Goal-setting

## Abstract

**Background:**

Lifestyle factors playing a role in the development of late-life disability may be modifiable. There is a need for robust evidence about the potential for prevention of disability through behavior change interventions.

**Methods/design:**

This feasibility study involves the development, implementation and initial testing of a behavior change intervention in a naturalistic setting. A small-scale randomized controlled trial (RCT) will investigate the implementation of a goal-setting intervention aimed at promoting behavior change in the domains of physical and cognitive activity in the context of a community resource center for over-50s. Healthy older participants attending the center (*n* = 75) will be randomized to one of three conditions: control (an interview involving a general discussion about the center); goal-setting (an interview involving identification of up to five personal goals in the domains of physical activity, cognitive activity, diet and health, and social engagement); or goal-setting with mentoring (the goal-setting interview followed by bi-monthly telephone mentoring). All participants will be reassessed after 12 months. Primary outcomes are levels of physical and cognitive activity. Secondary outcomes address psychosocial (self-efficacy, mood, quality of life), cognitive (memory and executive function), and physical fitness (functional and metabolic) domains. Cost-effectiveness will also be examined.

**Discussion:**

This study will provide information about the feasibility of a community-based lifestyle intervention model for over-50s and of the implementation of a goal-setting intervention for behavior change, together with initial evidence about the short-term effects of goal-setting on behavior.

**Trial Registration:**

Current Controlled Trials ISRCTN30080637 (http://www.controlled-trials.com)

## Background

Older people are living longer and form a greater proportion of the population than ever before. It is important to identify ways of promoting good health and preventing, delaying, or reducing the severity of age-related cognitive and physical disability, in order to enhance or maintain independence, wellbeing, and quality of life (QoL) for older people and limit the social and economic burden of care and support [1,2].

Multiple factors are involved in the pathway to age-related cognitive and physical disability. While some are not readily modifiable (for example, Apo-E status), lifestyle factors offer potential for change [3]. Increased levels of cognitive activity (CA) and physical activity (PA), and engagement in socially-oriented leisure activity, are associated with maintenance of cognitive function and reduced risk of Alzheimer’s disease [4]. Increased levels of PA contribute to improved physical functioning and health, help to combat depression, and decrease the risk of falls and cardiovascular disease. Theoretically it is suggested that engaging in such activities creates greater cognitive reserve and hence greater resilience in relation to age-related brain pathology [5]. Since many older people are socially isolated and cognitively and physically under-active [6,7], there is an urgent need for robust evidence regarding the potential for effective health promotion and prevention with regard to lifestyle factors. It is now timely to begin to test interventions addressing these factors in randomized controlled trials (RCTs) with long-term follow-up [4,8].

The complex range of factors involved indicates a need to address multiple risk factors in an integrated manner [4]. PA and CA trials have typically involved practice of circumscribed skills for a defined period, producing improvements in trained skills but little evidence of transfer of gains or long-term behavior change. Lifestyle activities, in contrast, can be characterized as the active co-ordination of multiple complex cognitive and physical abilities with the function of attaining personally-meaningful goals. Increasing CA and PA through lifestyle activity can ensure that changes are integrated into everyday life, stabilizing functioning and improving ability to cope with future challenges to wellbeing.

We aim to establish the feasibility of an innovative approach to increasing CA and PA among over-50s, including ‘hard to reach’ groups, based on goal-setting. Setting oneself stimulating but achievable goals is an effective means of changing behavior [9], providing motivation to perform better or maintain effort. The social cognitive theory of health behavior change [10] posits that this is most effective where perceived self-efficacy is such that the individual believes it is possible to achieve the desired effects through his/her own actions, and where adopting different behaviors proves enjoyable and socially-rewarding and enhances self-worth. Behavior change is most likely where the environment supports implementation of the desired behavior [11] through availability of peer support and accessibility of facilities and opportunities that can be matched with personal goals, and provides a means of overcoming perceived obstacles. In line with social cognitive theory, we will examine the benefits of developing a context that provides social and material support for behavior change, and of using a goal-setting intervention, with or without follow-up mentoring aimed at boosting self-efficacy and encouraging problem-solving regarding obstacles to change, to optimize use of this resource compared to simple access to facilities. We hypothesize that goal-setting, especially when accompanied by ongoing mentoring, will optimize engagement, leading to increased CA and PA, with benefits for cognitive, physical, social and psychological functioning, health, and QoL.

## Method

### Design

**Figure 1:**
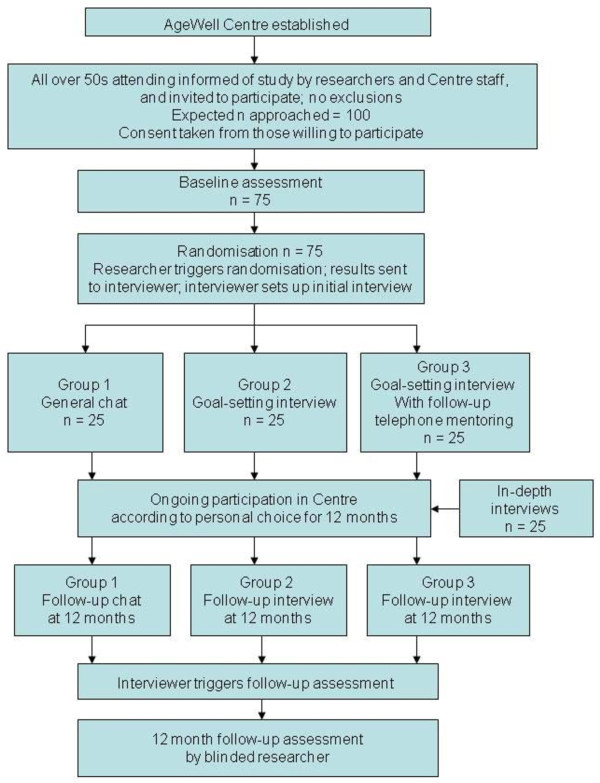
AgeWell Study CONSORT-style flowchart.

This is a feasibility study involving the development, implementation and initial testing of a naturalistic lifestyle intervention. A small-scale randomized controlled trial (RCT) will investigate the implementation of a goal-setting intervention aimed at promoting behavior change in the domains of PA and CA in the context of a community resource centre for over-50s. We will explore the acceptability of the approach (for example, willingness to participate and be randomized; attrition rates), its success in reaching those sections of the older population most in need and realizing its socially-inclusive ethos, and the age and demographic profile of those interested in engaging. We will examine the suitability of potential outcome measures, and use the findings to estimate critical parameters to inform the design of a large-scale RCT. A CONSORT-style flowchart is provided in Figure 1. The study has been approved by the relevant University and National Health Service Research Ethics Committees. This trial is registered with Current Controlled Trials, reference ISRCTN30080637.

### Participants

All individuals aged over 50 years living in the local community and attending the community resource center will be invited to participate, with no exclusions. If they do not wish to take part in research they may still attend the center, and in this case only records of attendance and activity participation will be taken. Sample size calculation for this feasibility study is based on anticipated attendance rates. We expect to randomize at least 75 individuals (target *n* = 25 per condition). Reasons for declining to participate will be recorded where given, although participants are not required to give this information. Attrition at any stage will be noted and reasons recorded where available, although participants are free to withdraw without giving a reason.

### Intervention

The intervention is conducted in partnership with Age Cymru Gwynedd a Môn (ACGM). Age Cymru is a registered charity within the devolved nation of Wales which forms part of Age UK, a major voluntary sector organization working to improve later life through provision of services and support throughout the UK [12]. ACGM provides services and support in the counties of Gwynedd and Anglesey in north-west Wales. The development of a community resource center for over-50s (‘AgeWell Centre’) will provide the context for the intervention, which compares the effects of three different styles of interview on subsequent behavior change.

The AgeWell Centre will be established in the village of Nefyn, Gwynedd, building on the experience of ACGM in setting up and running successful community-based facilities for over-50s in Anglesey, supported by a knowledge transfer partnership focused on physical activity, with several hundred beneficiaries over the past 2 years. A coordinator will facilitate the running of the center, supported by a volunteer management group of center attendees. The center will open 3 days a week to provide a range of activities that address the need for increased CA and PA and encourage adaptive health-related behavior around a central core of social interaction. Specific activities addressing the key areas in which behavior change is desired will be offered, targeting moderate to high intensity PA (walking, resistance training, tai chi) and CA (memory management, computing skills, reading group, creative writing, local history). Other activities will be developed and offered according to participant interest and need, for example, cooking for one, dancing, photography, gardening, community volunteering, and outings. Each activity will either be delivered by an outside facilitator or planned and carried out by a group of center participants. Participants will pay a nominal fee for all sessions, covering the costs of bringing in outside facilitators. The center will offer resources such as computers with internet access and fitness equipment. The center will also host a falls prevention group and offer a focus for community-based provision by other agencies. Records of attendance and participation for all those coming to the center will be compiled using the CharityLog database.

Those center attendees willing to participate in the RCT will be randomly allocated using a sequentially-randomized dynamic adaptive computer algorithm developed by the trials unit (NWORTH) and incorporating stratification by gender to one of three conditions following initial assessment [13]. Each condition involves a one-to-one interview with an appropriately-trained professional, lasting up to 1.5 h, as follows:

1. Group 1, control, will have a general discussion about the facilities and activities available.

2. Group 2, goal-setting, will have a structured goal-setting interview using the Bangor Goal-Setting Interview. The interview will explore current functioning in relation to PA and CA, as well as considering social engagement, diet, and health. The interviewer will have access to key information from the initial assessment such as details of identified health risks. Areas where the participant would like to make changes or improvements will be identified and prioritized, and up to five specific, realistic and achievable goals will be identified. Examples of goals might be increasing walking to 30 min per day, or learning to use email to communicate with a friend. Current performance, satisfaction with performance, and degree of confidence in carrying out the activity, will be rated by the participant on a scale of 1 to 10. Behavioral indicators of partial (25%, 50%, 75%) and complete goal attainment will be established for each goal. Ways in which the goal may be addressed will be discussed and linked to center activities (for example, walking group, computer skills sessions) or external facilities (such as, GP consultation, smoking cessation group), and a written individual action plan will be prepared.

3. Group 3, goal-setting with mentoring, will receive the same interview as Group 2 plus a follow-up mentoring phone call from the interviewer after 1 month (in month 2 of participation in the study) and then bi-monthly thereafter (in months 4, 6, 8, and 10) to review progress, problem-solve regarding any obstacles to progress, encourage and reinforce successes, and support maintenance of change. Each participant will remain in the study for 12 months, and will engage according to personal choice in center and related activities. Attendance and activity participation will be recorded. All participants will be reassessed after 12 months by a researcher blind to group allocation. At follow-up assessment, participants will re-rate performance and satisfaction with regard to each identified goal, and a rating of goal attainment will be made.

The Bangor Goal-Setting Interview, developed by the research team, provides a structured format for the goal-setting process. This interview is similar in some respects to the format of the Canadian Occupational Performance Measure [14], a well-established clinical method for measuring performance in a standardized manner across different individual goals [15], which has proved to be a sensitive measure of change in previous rehabilitation research [16]. However, it derives from a different theoretical model, incorporates a number of different features, and has been developed primarily as a research tool. The Bangor Goal-Setting Interview is based on the social cognitive theory of behavior change [10] and on the concept of motivational interviewing [17]. The interview structure allows researchers to select areas of behavior that are relevant to the aims of the study; for the present study we have selected the four areas of physical activity, cognitive activity, diet and health, and social engagement. The interview proceeds in three stages. Firstly, each of these areas is discussed in turn with a view to eliciting issues that might form the basis for behavioral goal-setting. For each area, the participant rates perceived importance of making changes in this area, and readiness to make changes in this area, on a scale of 1 to 10 (where 1 is not at all important/not at all ready and 10 is extremely important/completely ready). Once all areas have been discussed, the second stage involves revisiting each area in turn and negotiating specific behavioral goals that conform to SMART principles (specific, measurable, achievable, realistic, and time-delineated). For this study, we specified that two to five goals should be set, and that these could be in any of the four areas covered. Once a goal is set, and current performance described, possible barriers and facilitators to achieving the goal are discussed using specific prompts, with an emphasis on identifying the resources available to support behavioral change. Additionally, goal attainment indicators are specified, providing clear descriptors of what would constitute 25%, 50%, and 75% goal attainment. In the final stage of the interview, the participant is asked to rate, for each of the goals that have been identified, current performance and satisfaction with performance on a scale of 1 to 10 (where 1 is unable to perform/extremely dissatisfied and 10 is able to perform perfectly/extremely satisfied). Mean scores for performance and satisfaction with performance across goals are calculated by dividing in each case the sum of the scores for all goals identified by the number of goals set. At follow-up, the participant re-rates current performance and satisfaction with performance for each goal so that changes in ratings can be examined. The interviewer elicits information about current performance and uses the previously-specified goal attainment indicators to determine the extent of progress towards achieving the goal. Within the interview schedule there is scope to elicit informant ratings of performance and descriptions of goal attainment, for comparison purposes; this is particularly relevant for example where respondents have cognitive impairments. Similarly, assessor ratings can be made where the assessor has been involved in a therapeutic or supportive capacity in helping the participant work towards selected goals. As the present study involves healthy older participants, only self-ratings will be elicited.

### Measures

#### *Background measures taken at initial assessment only*

Demographic information including age, marital status, ethnicity, socio-economic status, and education.

Lifetime of Experiences Questionnaire (LEQ) [18]. The LEQ assesses the extent of complex mental activity undertaken across the lifespan. It is a 42-item scale with a mix of 5-point Likert scale and free responses which are coded into ordinal scale scores according to structured scoring principles. The LEQ asks about participation in educational, occupational and cognitively-stimulating lifestyle activities during young adulthood, mid-life and later life, and about engagement in mental activities that are not specific to a given life-stage.

## Measures taken at initial assessment and 12 month follow-up

### Primary outcomes

Florida Cognitive Activities Scale (FCAS) [19]. The FCAS is a 25-item scale examining the degree of current participation in a range of cognitively-stimulating activities. Participation in each activity is rated on a 6-point scale from 0 (have never done this activity/have not done this activity in the past year) to 4 (do this activity every day). Possible scores range from 0 to 100 with higher scores indicating greater involvement in activities. The total score will represent the primary outcome. Two subscale scores can also be calculated to provide additional information. The Higher Cognition subscale contains 10 items relating to engagement in cognitively challenging activities, with possible scores ranging from 0 to 40. The Frequent Activities subscale contains eight items describing everyday tasks or activities, with possible scores ranging from 0 to 32. We will use the FCAS to assess participation in CA during the previous month.

Physical Activities Scale for the Elderly (PASE) [20]. This 10-item scale investigates frequency of participation in a range of physical activities including sedentary activities, walking, light, moderate, or strenuous sport or recreational activities, strength and endurance exercises, light or heavy housework, home repairs or gardening, and caregiving, and the physical demands of any work undertaken for pay or as a volunteer. Response formats are three-point (seldom, sometimes, often) or four- point (<1 h, 1–2 h, 2–4 h, >4 h) scales or categorical (yes/no) responses. The total PASE score is computed by multiplying the amount of time spent in each activity (hours/week) or participation (yes/no) in an activity by the empirically derived item weights presented in [20] and summing over all activities. A higher total score indicates greater levels of activity.

### Secondary outcomes

#### *Assessment of psychosocial factors*

General Self-Efficacy Scale (GSES) [21]. This 10-item scale assessing self-efficacy presents a series of statements which the respondent rates on a 4-point scale to indicate how accurately the given statement describes him/her from 1 (not at all true) to 4 (completely true). Possible scores range from 1 to 40 with higher scores indicating higher perceived self-efficacy.

Center for Epidemiologic Studies Depression Scale (CES-D) [22]. This 20-item scale presents a list of feelings or behaviors and asks the respondent to rate the extent to which they felt or behaved this way during the past week on a 4-point Likert scale from 0 (rarely or none of the time) to 3 (most or all of the time). Possible scores range from 0 to 60 with higher scores indicating more signs of depression.

CASP-19 [23]. This 19-item scale assessing quality of life in older people is based on a needs satisfaction model and items relate to the four domains of control, autonomy, self-realization, and pleasure. Responses are scored on a four-point scale from 0 (never) to 3 (often). Possible scores range from 0 to 57 and higher scores indicate better perceived quality of life.

Social networks, social support, and social engagement: participants are asked about their frequency of contact with others including family and friends, about the quality of social relationships (rated on a scale from ‘very close’ to ‘not at all close’) and about their level and frequency of societal involvement including social and political engagement, participation in social recreational activities, cultural activities and leisure time such as holidays and trips. Items were taken from the English Longitudinal Study of Ageing [24]. With regard to social networks, people are considered disadvantaged if they do not live with a partner and do not meet any of their children, family or friends at least three times a week. With regard to social support, people are considered disadvantaged if they have no-one (partner, children, family, or friends) strongly supporting them. With regard to social engagement, a person is classified as ‘socially detached’ if there is disadvantage on three of six indicators of social participation: social networks, social support, social/political involvement, and participation in cultural, recreational, and leisure activities.

#### *Cognitive assessment*

Montreal Cognitive Assessment (MOCA) [25]. The MOCA is a brief cognitive screening test covering the domains of immediate and delayed recall, attention, executive function, visuospatial processing, language and naming, abstraction, and orientation. The total score can be corrected for level of education. A score of 26 or more out of 30 suggests no difficulties with cognitive function.

California Verbal Learning Test (CVLT-II) [26]. A list of words is read and the participant is asked to say all that s/he can remember. The same list is read, and recall elicited, four more times (five trials altogether). After trial 5 recall, a different list is read and the participant is asked to say all that s/he can remember. Then short-delay free recall of the first list is requested. After a 20 min delay (during which other tasks can be undertaken) long-delay free recall of the first list is tested. The total immediate recall score for List A trials 1 to 5 and the long-delay free recall score are the scores to be taken for this study.

Delis-Kaplan Executive Function System [27]. In this study we will use two of the d-KEFS subtests, Trail Making and Verbal Fluency. Trail Making is a timed visual-motor task assessing flexibility in thinking. The participant is first asked to draw a line connecting numbers spread randomly on the sheet of paper in ascending order. Next, the participant is asked to draw a line connecting numbers and letters in an alternating manner, with numbers in ascending order and letters in alphabetical order (1 - A - 2 - B - 3 and so on). The score to be used in this study is the time taken to complete the second task minus the time taken to complete the second task, in seconds. Verbal Fluency is a task assessing the ability to produce words according to a given set of rules and within a time limit. The participant is asked to produce as many different words as possible beginning with the letters f, a, and s. One minute is allowed for each letter. There should be no proper nouns or repetitions. The score is the total number of correct words summed across the three letters. Scaled scores can also be calculated.

#### *Physical fitness, health, and dietary assessment*

Health assessment covering medical history, anthropometric data, medication use, and blood pressure. A blood sample will be taken for lipid profile, to give a QRISK2 score [28]. This multifactorial score gives a percentage risk for cardiovascular disease over a 10-year period. The QRISK2 has been independently validated [29] and is based on data specific to the UK allowing for differing ethnicity and using postcodes to account for deprivation. The aim is to identify asymptomatic people without established cardiovascular disease (CVD) but who have a combination of risk factors which puts them at high total risk (estimated CVD risk ≥ 20% over 10 years) of developing atherosclerotic CVD for the first time. Blood samples will be analyzed by independent National Health Service laboratory staff. The blood results are copied to the participant’s primary care physician and also screened by JVH. Health needs (for example, regarding cardiovascular risk reduction) will be identified and details will be available to support the goal-setting interview process. If screening indicates a need for urgent medical attention in the view of the physician (JVH), an appropriate referral will be made.

Senior Fitness Test (SFT) [30]. Several brief subtests from the SFT will be used, involving common activities such as getting up from a chair, walking, lifting, bending, and stretching. These provide an assessment of upper and lower body strength, flexibility, and agility.

Physical fitness will be evaluated by determining predicted aerobic capacity from a submaximal graded exercise step test [31].

Mediterranean Diet Adherence Screener (MEDAS) [32]. This 14 item questionnaire examines participants’ adherence to a typical Mediterranean diet in terms of food intake habits and the frequency of consumption of foods such as olive oil and pulses. Adherence to this type of diet has been related to better physical and mental health outcomes [33].

#### *Process measures*

Data on attendance at the Centre and participation in Centre activities will be examined for participants in all three groups.

For groups 2 and 3, changes in goal performance and satisfaction ratings, and the extent of goal attainment, will be assessed.

### Measures for evaluation of cost-effectiveness

EQ-5day [34]. The EQ-5day is a standardized measure of health status and health outcome, applicable to a wide range of health conditions. In the first section, the respondent is asked to select one of three options for each of five dimensions: mobility, self-care, usual activities, pain/discomfort, and anxiety/depression. For each dimension, the three response options are coded on a 3-point scale from 1 (no problems) to 3 (unable to perform/extreme problem). This yields a descriptive profile of scores across the five dimensions. The second part of the measure is a visual analogue scale for self-rating of health-related quality of life (‘your health state today’).

ICECAP-O [35,36]. The ICECAP-O (ICEpop CAPability measure for Older people; ICEpop refers to Investigating Choice Experiments for the Preferences of Older People; http://www.epi.bris.ac.uk/research/programmes/icepop.htm) is a brief 5-item quality of life measure for older people, conceptually linked to the capability approach whereby wellbeing is defined in terms of an individual’s ability to ‘do’ and ‘be’ the things that are important in life. The five items, covering the attributes of attachment, security, role, enjoyment, and control, are each scored on a 4-point scale. Possible total scores range from 5 to 20 with higher scores indicating better perceived quality of life.

Client Services Receipt Inventory (CSRI) [37]. The CSRI asks respondents about their use of health and social care services. At initial assessment the focus will be on service use in the preceding 3 months. At follow-up the focus will be on service use over the past 12 months. The questions cover contact with a range of health and social care professionals, prescription of medications, hospital appointments and stays, participation in local authority funded activities such as day centers, and participation in activities run by voluntary organizations (other than the AgeWell Centre).

### Qualitative inquiry

In-depth qualitative interviews with a sub-sample of participants from all three conditions (target *n* = 25) will examine sociobiographical influences on processes of change.

### Procedure

Individuals attending the Centre will be given information about the study and invited to consider participating in the trial by a member of the research team, after which they will be given as much time as they need to decide whether they wish to take part. Those who consent to participate will complete the baseline assessment in two face-to-face sessions with a researcher, one focusing on psychological, social and cognitive measures and lasting approximately 1.5 h, and the other focusing on physical health and fitness and lasting approximately 1 h. The researcher will then trigger the randomization. Participants will be allocated to one of the three conditions and will engage in the interview conducted according to the protocol for their allocated condition. Those in the goal-setting with mentoring group will have bi-monthly mentoring phone calls from the interviewer over the following 12 months while the remainder will have no such contact. After 12 months all participants will engage in a follow-up interview in accordance with the condition to which they were allocated; for participants in the goal-setting and goal-setting with mentoring groups the interviewer will use the Bangor Goal-setting Interview to elicit current ratings of performance and satisfaction with performance for the originally-identified goals, while those in the control group will have a general discussion with the interviewer. The interviewer will then trigger the follow-up assessment and all participants will be reassessed by blinded members of the research team.

### Data analysis

Analysis of covariance (ANCOVA) will be used to examine CA and PA at follow up, co-varying for baseline levels. Two contrasts will be examined: a comparison between Group 1 and Groups 2 and 3 combined, addressing the question of whether goal-setting is effective in increasing CA and PA, and a comparison between Group 2 and Group 3, addressing the question of whether goal-setting supplemented with mentoring is more effective than goal-setting alone. The results will provide information about effect sizes and variability of response which will be needed for the design of a definitive trial. Data from the secondary outcome measures will be examined to inform the selection of measures for a definitive trial. QRISK2 scores will be compared to examine whether they provide confirmatory evidence of behavior change. Health economic analyses will be conducted to inform modeling for the definitive trial: primary cost-utility analysis; secondary cost-consequence analysis; cost-effectiveness acceptability curve. Background data and process measures will be examined to inform our understanding of the likely sample characteristics which would be encountered in a definitive trial, allowing us to consider variables that may have important influences on outcome or on the process of participation and prepare a comprehensive analysis plan.

Qualitative interview data, analyzed using a biographical narrative method, will contribute a deeper understanding of participants’ expectations and reasons for participating, the meanings they attach to health-related behavior, and the processes within which they actively engage (or not) with the challenges of aging [38]. These data will help to further develop and refine the intervention.

## Discussion

This study will provide information about the feasibility of a community-based lifestyle intervention model for over-50s and of the implementation of a goal-setting intervention for behavior change, together with initial evidence about the short-term effects of goal-setting on behavior. The study will help to determine whether this community-based lifestyle intervention improves cognitive and physical activity, health and well-being over a 12-month period. The findings are intended to support the selection of outcome measures and estimation of critical parameters for a larger-scale RCT.

A key element of the study is the development and adaptation of effective approaches to goal-setting and outcome measurement, and the application of these in a naturalistic setting. The study will be conducted in an inclusive, real-life context, allowing participants to integrate benefits directly into their daily lives. The integral involvement of older participants in shaping and managing the resources offered as part of the study will contribute to the development of social capital in the local community.

There is a risk that studies of this kind attract participation primarily from more socio-economically advantaged groups. This study is located in an area where lower socioeconomic groups are relatively strongly represented in the population and is conducted in partnership with an organization that has a strong track-record of accessing older people from across the full spectrum of socioeconomic advantage. This is a rural area where the population is relatively sparse and this presents particular challenges regarding transportation and accessibility. While it is possible that the choice of a rural area may affect the applicability of the findings to urban areas, the selection of a relatively disadvantaged area where facilities are sparse and transport links limited presents a demanding test of the feasibility of our approach.

The study will allow us to establish a demographic profile of participants and provide an understanding of who attends the Centre and why. It will also allow us to examine the extent to which the approach taken in the study is acceptable to people attending the Centre. While Centre attenders do not have to give a reason for either declining participation or withdrawing from the study, every effort will be made to obtain feedback on reasons for making such decisions. We anticipate that there will be more female than male attendees and randomization has been stratified to take account of gender. It will also be valuable to identify the age profile of attenders and if appropriate age will be taken into account in the statistical analysis.

The study findings will contribute to establishing an effective approach to promoting mental and physical health, enhancing mobility and independence, improving well-being and QoL, and identifying an effective means of preventing, delaying, or reducing the impact of, age-related disability and disease. The research integrates psychological, social, physiological, medical, epidemiological, public health, and economic perspectives, making it possible to consider the complex interactions between factors operating at each of these levels to determine health and wellbeing in later life.

This feasibility study contributes to establishing the effectiveness of lifestyle interventions in promoting healthy aging, preventing or delaying the onset of disability, and reducing costs of health and social care. Current knowledge is at a stage where we can begin to develop interventions addressing these aims [4], and this study will provide much-needed evidence to inform policy and support the development of approaches that can eventually produce benefits at population level.

### Trial status

This trial is currently recruiting. Recruitment commenced on 02 January 2012 and is scheduled to continue until 30 September 2012.

## Competing interests

The authors declare that they have no competing interests.

## Authors’ contributions

LC: Study concept, study design, preparation and drafting of study protocol, development of goal-setting interview, drafting of manuscript. JVH: Study design, preparation of study protocol, review of manuscript. IRJ: Study design, preparation of study protocol, review of manuscript. JMT: Study design, preparation of study protocol, review of manuscript. SMN: Trial management, development of study protocol, recruitment and assessment of participants, review of manuscript, development of goal-setting interview. BH: Study design, preparation of study protocol, review of manuscript. CJW: Study design, preparation of study protocol, statistical analysis plan, review of manuscript. All authors read and approved the final manuscript.
